# Application of 3D printed patient-specific instruments in the treatment of large tibial bone defects by the Ilizarov technique of distraction osteogenesis

**DOI:** 10.3389/fsurg.2022.985110

**Published:** 2023-01-06

**Authors:** Hao Zheng, Lili Wang, Wenbo Jiang, Ruiqing Qin, Zhiyu Zhang, Zhuqing Jia, Jian Zhang, Yong Liu, Xuejian Gao

**Affiliations:** ^1^ Department of Trauma Surgery, Affiliated Hospital of Weifang Medical University, Weifang, China; ^2^ School of Clinical Medicine, Weifang Medical University, Weifang, China; ^3^ Clinical and Translational Research Center for 3D Printing Technology, Shanghai Ninth People’ s Hospital, Shanghai Jiao Tong University School of Medicine, Shanghai, China

**Keywords:** 3D printing, patient-specific instruments, bone defects, distraction osteogenesis, Ilizarov ring fixator

## Abstract

**Background:**

The Ilizarov technique of distraction osteogenesis is an effective treatment for tibia defect. However, repeated attempts to reduce due to the complexity of the bone defect during the operation will increase the operation time and iatrogenic injury, and excessive radiation exposure. Three-dimensional (3D)-printed patient-specific instrument (PSI) for preoperative 3D planning and intraoperative navigation have the advantages of accuracy and visualization. The purpose of this study is to investigate whether 3D-printed PSI is helpful to correct tibial bone defects accurately and effectively.

**Method:**

From May 2019 to September 2022, 19 patients with tibial bone defects were treated, including 9 males and 10 females, aged 37 to 64 years. There were 4 cases in proximal tibia, 9 in midshaft tibia and 6 in distal tibia. All were treated with Ilizarov technique of distraction osteogenesis. 3D-printed PSI was used in 9 cases, while traditional surgery was used in 10 cases. All patients underwent computed tomography before surgery. Computer software was used to analyze the measurement results, design and print PSI. During the operation, PSI was used to assist in reduction of tibia. Operation times were recorded in all cases, the number of fluoroscopy during the operation, and the varus/valgus, anteversion/reversion angle after the operation were measured. All measurement data were expressed by means ± SD, and Student's t test was used to examine differences between groups. The chi square test or Fisher's precise test was used to compare the counting data of the two groups.

**Result:**

All PSI matched well with the corresponding tibia bone defect, and were consistent with the preoperative plan and intraoperative operation. The affected limb had a good reduction effect. The operation time from the beginning of PSI installation to the completion of Ilizarov ring fixator installation was 31.33 ± 3.20 min, while that in the traditional operation group was 64.10 ± 6.14 min (*p* < 0.001). The times of fluoroscopy in the PSI group during operation was 10.11 ± 1.83, and that in the traditional operation group was 27.60 ± 5.82. The reduction effect of tibia in PSI group was better than that in traditional operation group, with the average angle of PSI group is 1.21 ± 0.24°, and that of traditional operation group is 2.36 ± 0.33° (*p* < 0.001).

**Conclusion:**

The PSI simplifies procedures, reduces the difficulty of the operation, improves the accuracy of the operation, and provides a good initial position when used in distraction osteogenesis to treat the tibial defects.

## Introduction

Trauma, infection and malignancy can lead to bone loss (1). Most fractures or bone defects heal spontaneously without major interventions due to bone's intrinsic capability of regeneration. However, large bone defects lack the ability for self-regeneration and require open reduction (2–5). The tibia is one of the most common positions in long bone defects. Therefore, good reduction and functional reconstruction are of great significances to improve the chances of a successful outcome.

Ilizarov ring fixator is a very universal and powerful device. The distraction osteogenesis can eradicate infection, compensate bone defects and promote bone healing through progressive bone histogenesis. Besides, it can correct deformities and limb length differences and has become an effective method to treat segmental bone defects (6–11). This technique is challenging for orthopedic surgeons because the Ilizarov ring fixator cannot compensate for poor technique or poor reduction (12). Accurate reduction of the tibia during operation is the premise to control the correction angle of each dimension. However, due to the bone defect, the affected limb can move freely, which means the operator needs to adjust and reset the tibia repeatedly according to the general appearance during the operation. Therefore, the effect of tibial orthopedics during operation is often not ideal, leading to unsatisfactory surgical results.

3D printing is an additive manufacturing (AM) technology capable of producing anatomical matching and patient specific devices and structures with high adjustability and complexity that previously have been extremely difficult (13, 14). With the rapid development of 3D printing technology in the medical field, 3D printing technology plays a vital role in orthopedic surgery planning. Patient specific instrumentation (PSI) produced by 3D printing shows high accuracy and personalization in orthopedic repair and reconstruction, and has achieved good clinical results (15–19). For this reason, we conducted a single-center retrospective study on patients with tibial bone defects admitted from May 2019 to September 2022. The purpose of this study was to explore whether the PSI with personalized 3D printing could provide accurate and effective reduction of the tibia and a good initial position for future distraction osteogenesis.

## Materials and methods

### General information

There were 9 males and 10 females, ranging in age from 37 to 64 years, with an average age of 49.8 years. There were 4 cases in proximal tibia, 9 in midshaft tibia and 6 in distal tibia. Among all the patients, 12 cases were caused by traffic accidents and 7 cases were caused by heavy pound. Patients that fulfilled the following criteria were eligible for the study: (i) the patient without serious cardiopulmonary insufficiency, osteoporosis and other related surgical contraindications, (ii) has good compliance, and (iii) complete clinical data. Patients were excluded from the study when (i) the patient suffered from serious postoperative complications, (ii) other fractures were present, (iii) articular surface defects, and (iv) the patient with pathological fracture and bone tumor. All patients were treated with distraction osteogenesis. Ilizarov ring fixator was installed in 10 patients (2 cases in proximal tibia, 4 cases in midshaft tibia and 4 cases in distal tibia) with the help of 3D-printed PSI for reduction and correction of tibial fracture. 9 cases (2 cases of proximal tibia, 5 cases of midshaft tibia and 2 cases of distal tibia) underwent traditional surgery. Before surgery, all patients underwent imaging examination and computed tomography (CT) scan.

This study was approved by the ethics committee of the Affiliated Hospital of Weifang Medical University and carried out in accordance with the ethical standards of the declaration of Helsinki (revised in 2013). All patients and their families received sufficient information about the study and signed informed consent.

### Preoperative planning and fabrication of 3D-printed PSI

The Medical 3D Printing Innovation Research Center of Shanghai Jiaotong University and the Department of Trauma Surgery in the Affiliated Hospital of Weifang Medical University participated in the design of PSI, which was fabricated by Blackstone Intelligent Manufacturing Research Institute (Shandong) Co., Ltd. Imaging the tibia in detail is an important first step in generating 3D-printed PSI. First, the affected limb of each patient was scanned with 64-row spiral CT to obtain images including the tibia and fibula (1.0 mm; 120 kV). DICOM data sets were acquired to generate DICOM files, which was processed by medical certified Image Processing Software (Mimics®, Materialise, Leuven, Belgium) and then a 3D bone model was generated. The line for osteotomy was determined by the size and location of sequestrum. The healthy contralateral tibia was mirrored and used as a reconstruction template. Through the iterative closest point (ICP) algorithm, the mirrored bone model was superimposed on the affected tibia to reconstruct the tibial anatomy. Subsequently, the PSI was simulated and designed in accordance with the anatomical structure of the tibia.

### Guide design

**Figure 1:**
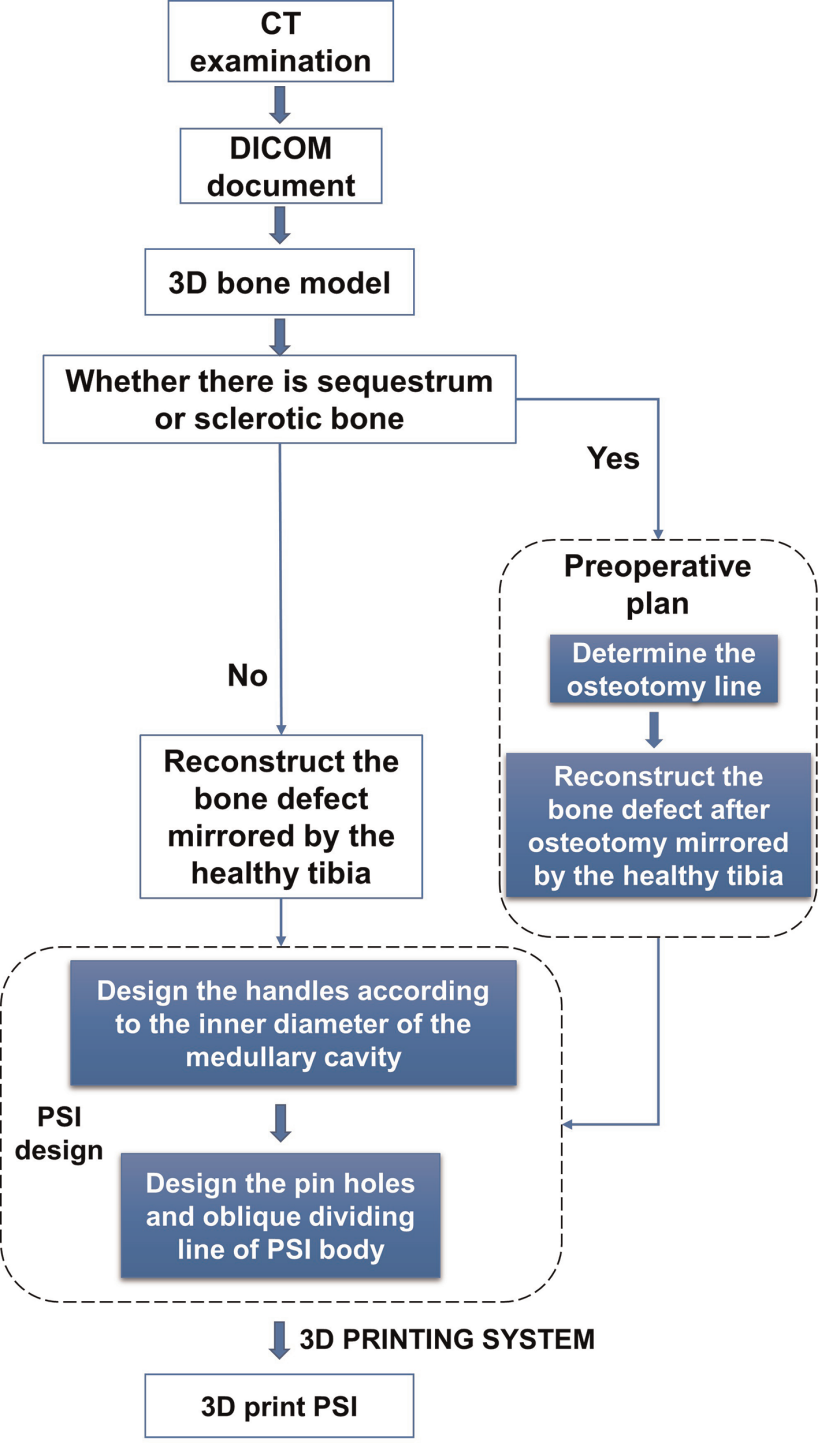
The design procedure of PSI.

The PSI is a 3D smooth interface structure composed of a body and handles at both ends. The diameters of the handles at both ends are respectively matched with the proximal and distal medullary cavity of the tibial defect. The handles can be smoothly inserted into the proximal and distal medullary cavity, so that the PSI can be fixed to the remaining tibia stably. Since the PSI has handles at both ends, it cannot be placed in the bone defect. Therefore, we designed oblique dividing lines and two rod holes perpendicular to the sagittal plane in the body of PSI, which can separate the PSI into two parts and both parts have two rod holes respectively, and rods corresponding to the inner diameter of the rod holes were designed for assembly and disassembly of PSI. PSI was printed with photosensitive resins using 3D PRINTING SYSTEM (SLA; China). PSI is sterilized by cold gas sterilization technology (hydrogen peroxide) before utilization. The whole process from the confirmation of the operation plan to the production of PSI was completed within 3 days. The design procedure of PSI is shown in Figure 1.

### Surgical method

The surgical methods of the patients were as follows. The patient was placed in supine position with the affected limb padded up and under general anesthesia. The surgical approaches were designed according to the condition of soft tissue defect. All the inactivated tissues were removed with the two broken ends cleaned, and the sclerotic bone was removed along the osteotomy line designed before the operation. The assistant pulled the affected limb and inserted the distal and proximal handles of PSI into the medullary cavity of the broken end respectively. Subsequently, the rods were inserted into the rod hole after the PSI was well fitted with the bone defect. PSI can temporarily maintain tibial reduction and normal length of affected limb to prevent limb rotation and axial deviation. Then, two 2.5 mm Kirschner wires were inserted into the proximal and distal rings to fixed the ring fixator. The middle sliding bone segment was fixed with a threaded half needle. In this step, we performed a 1 cm incision and carefully protected the skin, subcutaneous tissues and periosteum. Last, we tightened the nut to complete the fixation of ring fixator. The osteotomy of the sliding bone segment was determined according to injured position and blood supply. Subsequently, we pulled out the rods and gently removed the PSI from the bone defect. Finally, the alignment of the tibia and the fixation of the ring fixator was examined based on x-ray. We judged the alignment by measuring the following two types of angles, varus/valgus, anteversion/retroversion, and we calculated the average angles of the two groups.

### Postoperative treatment

During the perioperative period, routine antibiotics, anti-inflammatory analgesics and other treatment measures were taken to avoid infection. The Kirschner wires were wrapped with alcohol gauze, and the gauze was changed every 2 days. Bone segment was transported at the rate of 0.25 mm/time and 4 times/day 5 days after operation. The patients should start functional exercise after detumescence and pain relief including isometric contraction of muscle group and functional activity of adjacent joints. Regular radiographs were taken to observe the bone transport length and the osteogenesis in the periosteum, and the extension scheme was adjusted according to the x-ray.

### Statistical analysis

Microsoft Excel 2019 was used to sort out the collected data of two groups of research objects and establish the original database. All statistical analyses were conducted using Stata software version 9.0 (StataCorp LP, College Station, TX, USA) and SPSS software version 26.0 (SPSS Inc., USA). All measured data were expressed as mean ± SD, and the difference between groups was tested by *t*-test. The chi square test or Fisher's exact test was used to compare the two groups of counting data.

## Results

Nine patients with tibial bone defects were treated with 3D-printed PSI. During the operation, PSI was fixed on the tibial medullary cavity through the handle system. The PSI and bone defects were completely matched. The operation results were consistent with the postoperative evaluation. The operation process was very smooth with the assistance of PSI. The time from the beginning of PSI installation to the completion of ring fixator installation was 31.33 ± 3.20 min. During the operation, the position and direction of the needle were determined by fluoroscopy, and the times were 10.11 ± 1.83. Postoperative anteroposterior and lateral x-ray films showed Varus/Valgus (1.32 ± 0.26°) and Anteversion/Reversion (1.11 ± 0.36°). The average angulation is 1.21 ± 0.24°. The Ilizarov ring fixator was fixed reliably without axial deviation.

Ten patients with tibial bone defects treated by traditional surgery, the time from the beginning of tibial reduction to the completion of ring fixator installation is 64.10 ± 6.14 min. During the operation, fluoroscopy was used to judge the tibial correction and determine the position and direction of the needle. The number of times of fluoroscopy is 27.60 ± 5.82. Postoperative anteroposterior and lateral x-ray films showed Varus/Valgus (2.68 ± 0.40°) and Anteversion/Reversion (2.04 ± 0.45°). The average angulation is 2.36 ± 0.33°. The ring fixator was firmly fixed without axial displacement. The operation time and intraoperative fluoroscopy times of patients receiving 3D-printed special instruments were significantly less than those of patients receiving traditional surgery (*p *< 0.001). The tibial angulation in the 3D-printed PSI group was significantly lower than that in the traditional operation group (*p *< 0.001). There was no difference in general conditions between the two groups (*p *> 0.05). The clinical characteristics and results were summarized in Table 1.

**Table 1:** Comparation of baseline characteristics and results of the PSI group and traditional surgery group.

Group Cases	Age (year, $\bar{x}\pm s$)	BMI	Gender		Position			Bone defect length (mm)	Measuring angle (degrees, °, $\bar{x}\pm s$)				Operation time (min, $\bar{x}\pm s$)	Fluoroscopy time ($\bar{x}\pm s$)
		M	F	Pro	Mid	Dis	Varus/Valgus		Anteversion/Retroversion	Average	Rotation			
PSI group	9	51.11 ± 9.52	21.97 ± 3.50	3	6	2	5	2	84.50 ± 33.88	1.32 ± 0.26	1.11 ± 0.36	1.21 ± 0.24	N/P	31.33 ± 3.20	10.11 ± 1.83
Traditional surgery group	10	54.20 ± 6.07	22.27 ± 3.98	6	4	2	4	4	70.50 ± 29.65	2.68 ± 0.40	2.04 ± 0.45	2.36 ± 0.33		64.10 ± 6.14	27.60 ± 5.82
*p*-value		0.406	0.863	0.370		0.836			0.350	<0.001***	<0.001***	<0.001***	** **	<0.001***	<0.001***

Operation time began with the installation of the ring fixator.

1. x-ray examinations were performed after the reduction and fixation process in order to confirm the reduction accuracy.

2. The data in this table is the average data obtained by five senior orthopedists from different medical institutions.

3. ***p < 0.001; Bold values mean statistically significant.

4. BMI Body Mass Index, M male, F female, Pro proximal tibia, Mid midshaft tibia, Dis distal tibia.

### Typical case report

**Figure 2:**
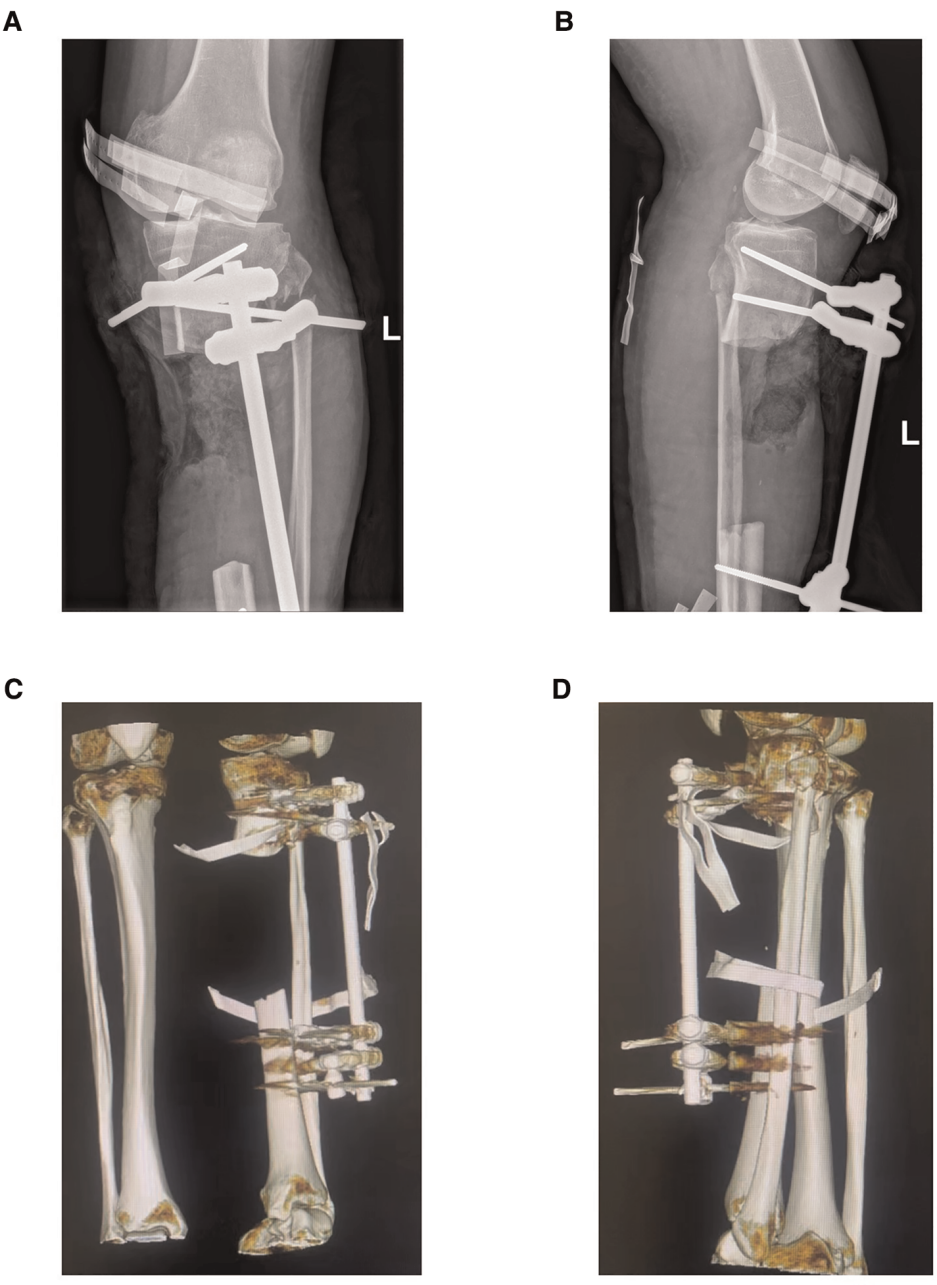
Preoperative x-ray and three-dimensional reconstruction of computed tomography (CT) of the left lower limb in case 1. (**A**) Preoperative x-ray on anterior-posterior (AP) view of affected limb in case 1, and there is an approximately 144 mm long bone defect at the proximal tibia. (**B**) Preoperative x-ray on lateral view of affected limb in case 1. (**C**,**D**) Three-dimensional reconstruction image of case 1 before operation.

**Figure 3:**
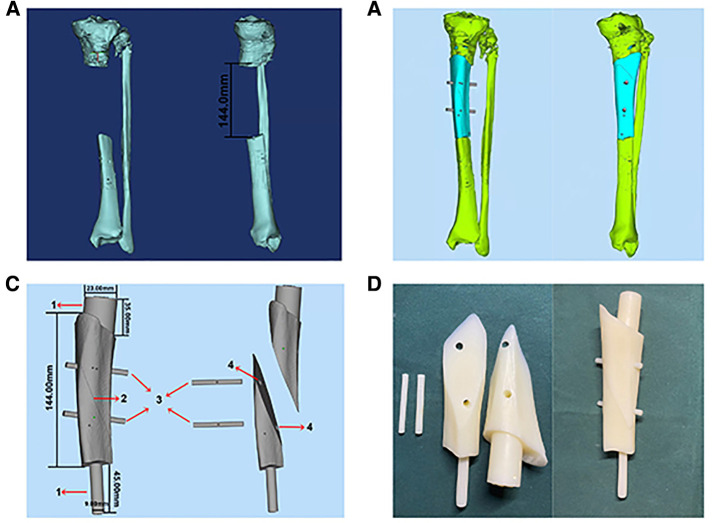
The design of PSI in case 1. (**A**) 3D bone model of affected limb in case 1, the length of bone defect is 144.0 mm. (**B**) 3D reconstruction of bone defect. (**C**) The design of PSI in case 1: (1) Handle. (2) Oblique dividing line. (3) Rod. (4) Rod hole. (**D**) 3D-printed PSI of case 1.

**Figure 4:**
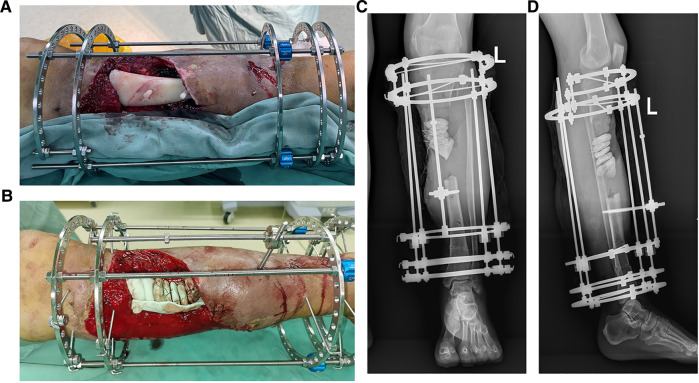
The intraoperative application of PSI and postoperative x-ray images in ilizarov technique of distraction osteogenesis in case 1. (**A**) The PSI was put into the bone defect to maintain the alignment of the fracture fragments. (**B**) Several pie-shaped bone cements were stacked into the defect which would be gradually withdrawn according to the bone transportation. (**C**) Postoperative x-ray on anterior-posterior (AP) view of case 1, the angulation of valgus is 1.74°. (**D**) Postoperative x-ray on lateral view after operation of case 1, the angulation of anteversion is 1.14°.

In case 1, a 61-year-old male patient was diagnosed with open comminuted fracture of left tibia and fibula. External fixator fixation was performed in local hospital. The patient and his family members were strongly willing to save limbs and were transferred to our hospital for further treatment on the same day. According to the results of x-ray and 3D-CT examination, the length of tibial bone defect was 144.00 mm which was showed in Figure 2. Under the suggestion of the surgeon, the patient chose to carry out the Ilizarov technology of distraction osteogenesis with 3D-printed PSI for tibia to repair and reconstruct the bone defect. Figure 3 shows the design of PSI and the 3D-printed PSI. The application of PSI and the bone cement (Refobacin® Bone Cement R, Biomet, France) mixed with vancomycin filled on the bone defect during the operation is showed in Figures 4A,B. After the operation, the wound was treated with vacuum sealing drainage (VSD), skin grafting in selective operation, and removement of the bone cement gradually according to the bone transportation. The x-ray film of the affected limb was taken after the operation which suggested the fracture fragment was in alignment. The angulation of valgus is 1.74°, and the angulation of anteversion is 1.14°. In addition, the Ilizarov ring fixator was stable (Figures 4C,D).

**Figure 5:**
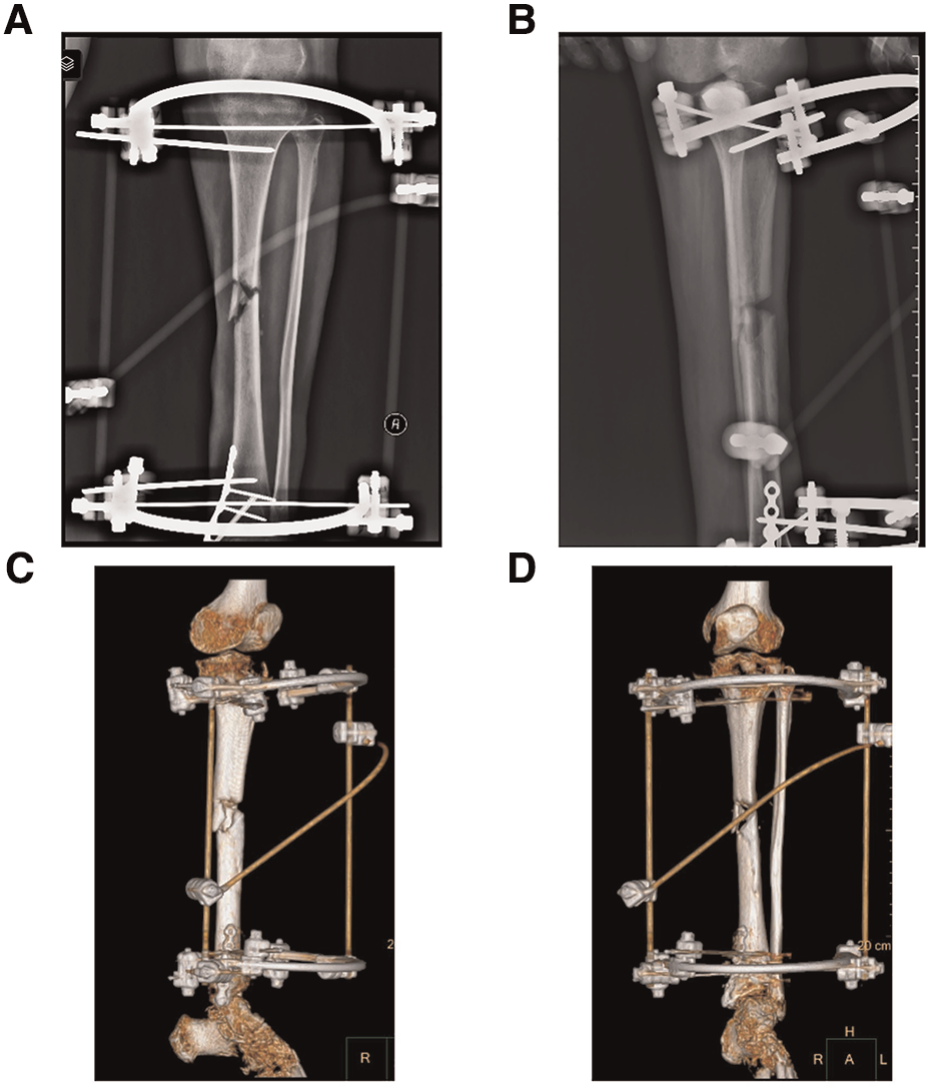
Preoperative x-ray and three-dimensional reconstruction of computed tomography (CT) of the left lower limb in case 2. (**A**) Preoperative x-ray on anterior-posterior (AP) view of case 2, and there is bone nonunion in the middle of tibia. (**B**) Preoperative x-ray on lateral view of case 2. (**C**,**D**) Three-dimensional reconstruction image of case 1 before operation.

**Figure 6:**
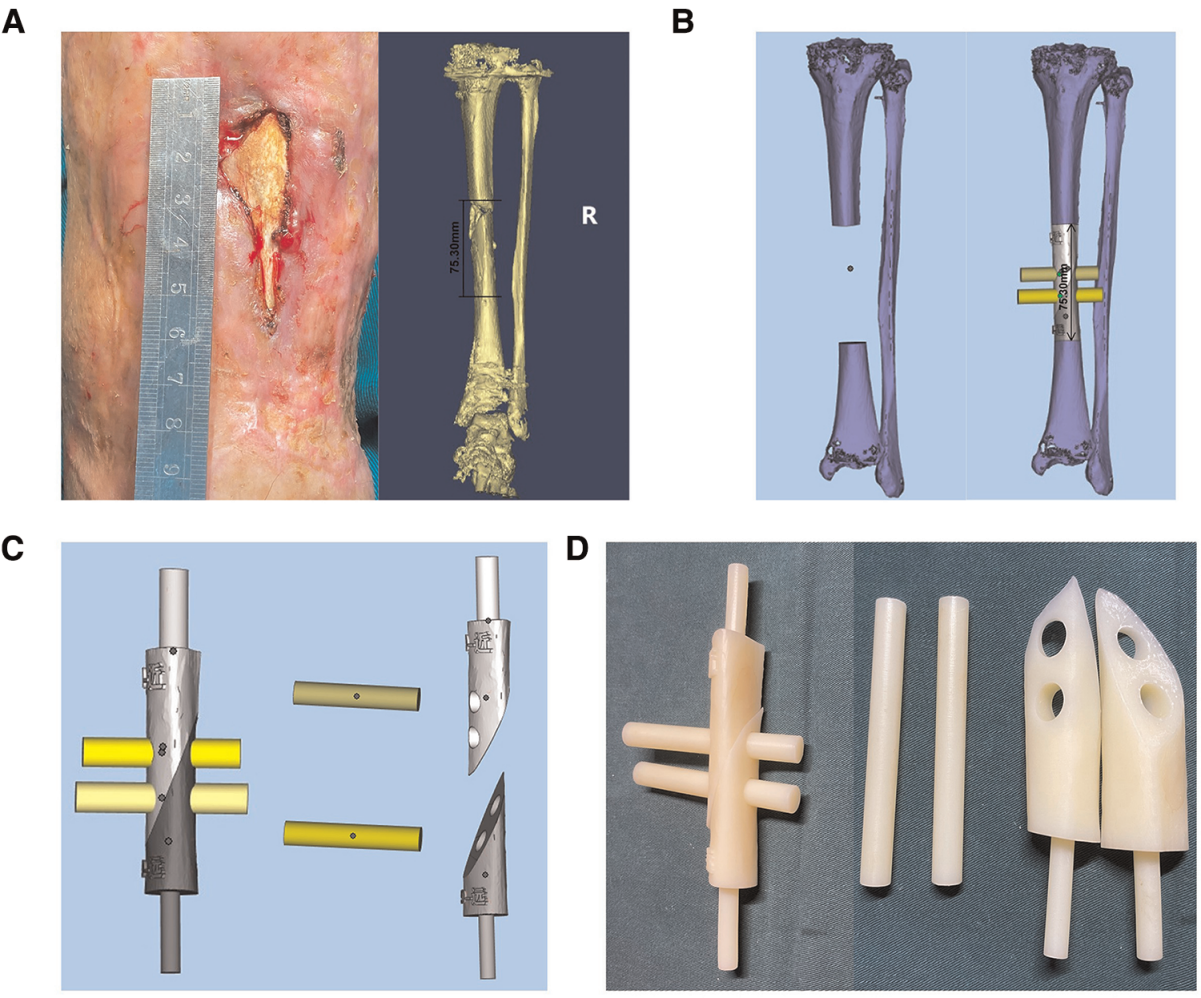
The exposure of sequestrum before operation and the design of PSI in case 2. (**A**) The patient's sequestrum was exposed before operation and the designed length of osteotomy was approximately 75.3 mm. (**B**) 3D bone model of affected limb in case 2, the length of bone defect is 75.30 mm. (**C**) The design of PSI. (**D**) The PSI of case 2.

**Figure 7:**
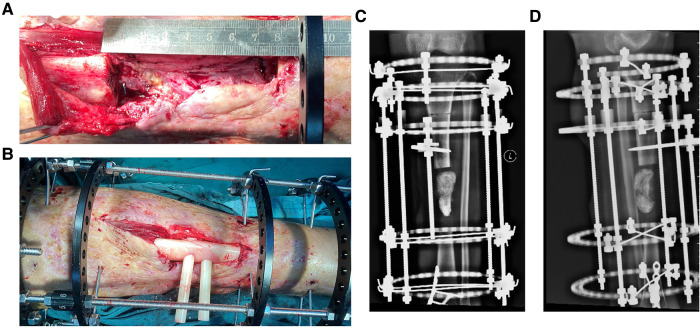
The osteotomy was carried and intraoperative application of PSI in Ilizarov technique of distraction osteogenesis and the postoperative x-ray image in case 2. (**A**) Intraoperative image of case 2 after osteotomy. (**B**) The PSI was put into the bone defect to assist reduction. (**C**) x-ray image on Anterior-posterior (AP) view after operation of case 2, and the iodoform gauze were filled in bone defect. (**D**) x-ray image on lateral view after operation of case 2.

In case 2, a 48-year-old female patient came to our hospital for further treatment because of bone nonunion along with bone exposure in the middle tibia 6 months after external fixation. Multiple treatment options were initially considered. (1) Bone transplantation: due to the large bone defect and the high risk of subsequent fracture. (2) Free bone tissue transplantation: the process is complex, limited by the size of bone defect, and has a high rate of complications on the donor area. (3) Amputation: the patient and his family members have strong desire for limb preservation. Through fully communicating with the patient and his family members, the surgeon decided to use 3D-printed PSI to assist tibial reduction and perform the Ilizarov technology of distraction osteogenesis. Preoperative x-ray and CT reconstruction images showed that the bone in the middle tibia was discontinuous and broken fragments were displaced partially (Figure 5). In order to ensure that fresh bone fragment is exposed and obtain adequate healthy soft tissue coverage, on the 3D bone model of the affected limb, an osteotomy line is designed to trim the two bone ends according to the condition of bone exposure and sequestrum, and the length of the bone defect is about 75.00 mm (Figure 6A). The design of PSI is showed in Figures 6B–D. In addition, the application of sequestrectomy and PSI during the operation is showed in Figures 7A,B. Iodoform gauze was used to fill the wound, which would to be removed one week after the operation. and the wound was sutured directly. The x-ray film of the affected limb taken after the operation showed that the alignment was good. The angulation of varus is 0.98°, and the angulation of retroversion is 0.55°. No complications such as loosening of fixed needle and infection of needle site were found (Figures 7C,D).

## Discussion

Accurate reduction is conducive to bone healing and functional recovery. A stable temporarily fixed reduction is a prerequisite for easy application of Ilizarov ring fixator with Kirschner wires and/or threaded half needle. However, at present, in the Ilizarov technique of distraction osteogenesis, the bone defect reduction mainly depends on the surgeon's experience, and the complexity of the operation limits its clinical application (20). Repeated attempts to use C-arm x-rays will increase the operation time and may cause new damage to the fracture site. Overexposure to x-rays will affect the health of surgeons and patients (21).

In recent years, 3D printing technology has become more and more popular in the medical field. PSI was developed as an alternative to navigation systems. In 1998, the first reported application in orthopedics was the production of a guide for auxiliary placement of pedicle screws in the spine (22). Afterwards, it gradually expanded to the guide for shoulder, hip, knee and ankle joint selective and trauma surgery, which can intuitively and effectively place the components in the required positions, and improve the accuracy of surgery, and shorten the operation time and fluoroscopy time (23–26). Although PSI is widely used in other operations, it is rarely used in correction and reduction of open fractures. How to achieve better alignment in the treatment of tibial bone defect is challenging. The surgery needs the assistance of PSI to improve the accuracy and effect of the operation and reduce complications and sequelae.

In this study, the specially designed PSI allows full resection of necrotic and sclerotic bone, and then is designed according to the specific situation of the bone defect after virtual reduction. Therefore, the PSI applying support force between the broken ends of the tibia can perfectly fit the defect site after placement, and relieve the traction of the soft tissue on the tibial stump, so that the tibial reduction can be maintained temporarily, and the tibia can obtain satisfactory reduction effect. Besides, the PSI provides the operator with a more convenient and rapid method of accurate reduction.

Under the application of PSI, the number of intraoperative fluoroscopy and the operation time were significantly shorter than traditional surgery group. The varus/valgus and anteversion/retroversion angles after tibial reduction were smaller than traditional surgery group. PSI reduces the difficulty of tibial reduction and simplifies the surgical process. In addition, PSI provides accurate initial position for distraction osteogenesis in the future, and reduce the possibility of postoperative complications caused by poor alignment of fracture fragments.

The clinical treatment experience shows that this new type of PSI has the following advantages in the treatment of tibial bone defects. First, it enables personalized patient data to be measured by computer to determine parameters such as osteotomy angle and plane at the fracture end. A lot of preparation work needs to be done before the operation, including image processing and PSI design and manufacturing. These steps are completed externally, which are not limited by the operation time and will not increase the pain and risk of the patient. Second, it avoids repeated operation during difficult reduction, which leads to the damage of surrounding tissues and bone, and reduces the incidence of complications. Third, the application of PSI is simple and convenient. PSI can be firmly fixed on the corresponding anatomical structure, accurately correct the tibia and maintain reduction, which saves the operation time and workload. Finally, the PSI can reduce the surgical challenges caused by difficult reduction, shorten the learning curve of new clinicians, and facilitate their surgical training.

The pre-operative design points of 3D-printed PSI applied in the distraction osteogenesis are as follows. First, attention should be paid to achieving good dimensional matching between PSI and bone defect after sequestrectomy. If the PSI is too small to fit well with the bone defect, the PSI will become loose and cannot achieve the expected mechanical stability, which will affect the effect of tibial reduction. Too large prosthesis will make it difficult for PSI to be inserted into the bone defect or need to remove more normal bone tissue, leading to the expansion of the bone defect and increasing the treatment risk of patients. Secondly, the PSI we designed is composed of body and handles. The body is fixed with oblique split nails and rods, which not only preserves the 1:1 accurate matching between the body and the anatomical structure of the bone defect, but also ensures that the PSI can be safely placed in the defect.

This study has several limitations. First of all, the sample size of the two groups is too small. Additionally, PSI is only used to correct the tibial force line during the operation and the process of bone transportation is affected by many factors. Therefore, it is necessary to expand the sample size and conduct long-term follow-up for the patients to further study the advantages and disadvantages of the treatment methods we proposed. Third, we did not conduct a precise cost-benefit analysis, which is important for a new technology. Cost-effectiveness analysis requires more data (e.g., direct costs, indirect costs, hidden costs), especially long-term follow-up, to judge the potential benefits of clinical outcomes (27–29).

## Conclusion

The PSI constructed by computer aided design and 3D printing technology has been successfully applied to the distraction osteogenesis of tibial bone defects. The instrument can simplify the operation procedure, reduce the operation time and fluoroscopy times, improve the operation accuracy, and shorten the learning curve of new clinicians. Therefore, this method has the value of popularization and application.

## Data Availability

The datasets generated during and/or analyzed during the current study are available from the corresponding author on reasonable request.
